# Association of the *FCN2* Gene Single Nucleotide Polymorphisms with Susceptibility to Pulmonary Tuberculosis

**DOI:** 10.1371/journal.pone.0138356

**Published:** 2015-09-17

**Authors:** Dan-Dan Xu, Chong Wang, Feng Jiang, Li-Liang Wei, Li-Ying Shi, Xiao-Mei Yu, Chang-Ming Liu, Xue-Hong Liu, Xian-Min Feng, Ze-Peng Ping, Ting-Ting Jiang, Zhong-Liang Chen, Zhong-Jie Li, Ji-Cheng Li

**Affiliations:** 1 Institute of Cell Biology, Zhejiang University, Hangzhou 310058, P.R. China; 2 Dongzhimen Hospital Affiliated to Beijing University of Chinese Medicine, Beijing 100700, P.R. China; 3 Department of Respiratory Medicine, The Sixth Hospital of Shaoxing, Shaoxing 312000, P.R. China; 4 Department of Clinical Laboratory, Zhejiang Hospital, Hangzhou 310013, P.R. China; 5 School of Medicine, Shaoxing University, Shaoxing 312000, P.R. China; 6 School of Laboratory Medicine, Jilin Medical College, Jilin 132013, P.R. China; St. Petersburg Pasteur Institute, RUSSIAN FEDERATION

## Abstract

Ficolin-2 (FCN2) is an innate immune pattern recognition molecule that can activate the complement pathway, opsonophagocytosis, and elimination of the pathogens. The present study aimed to investigate the association of the *FCN2* gene single nucleotide polymorphisms (SNPs) with susceptibility to pulmonary tuberculosis (TB). A total of seven SNPs in exon 8 (+6359 C>T and +6424 G>T) and in the promoter region (-986 G>A, -602 G>A, -557 A>G, -64 A>C and -4 A>G) of the *FCN2* gene were genotyped using the PCR amplification and DNA sequencing methods in the healthy controls group (n = 254) and the pulmonary TB group (n = 282). The correlation between SNPs and pulmonary TB was analyzed using the logistic regression method. The results showed that there were no significant differences in the distribution of allelic frequencies of seven SNPs between the pulmonary TB group and the healthy controls group. However, the frequency of the variant homozygous genotype (*P* = 0.037, -557 A>G; *P* = 0.038, -64 A>C; *P* = 0.024, +6424 G>T) in the TB group was significantly lower than the control group. After adjustment for age and gender, these variant homozygous genotypes were found to be recessive models in association with pulmonary TB. In addition, -64 A>C (*P* = 0.047) and +6424 G>T (*P* = 0.03) were found to be codominant models in association with pulmonary TB. There was strong linkage disequilibrium (r^2^ > 0.80, *P* < 0.0001) between 7 SNPs except the -602 G>A site. Therefore, -557 A>G, -64 A>C and +6424 G>T SNPs of the *FCN2* gene were correlated with pulmonary TB, and may be protective factors for TB. This study provides a novel idea for the prevention and control of TB transmission from a genetics perspective.

## Introduction

Tuberculosis (TB) is a chronic infectious disease caused by *Mycobacterium tuberculosis* (Mtb). Mtb can cause other kinds of TB, but pulmonary TB is the most common [1]. According to the World Health Organization’s 2013 global report on TB, an estimated 9.0 million people developed TB and 1.5 million died from this disease, 360,000 of whom were HIV-positive [2]. One-third of the world’s population is infected with Mtb, of which 5–10% will eventually develop active TB [3].

In 1926, newborn infants from the town of Lübeck in Germany were accidentally vaccinated with live Mtb instead of the vaccine Bacillus Calmette-Guérin (BCG). Some of the babies became severely ill and died, while others were unaffected [4]. In addition, a higher concordance rate has been found in monozygotic twins (60%) than dizygotic twins (20%) [4]. It therefore suggests that genetic factors can influence an individual's susceptibility to TB. Reported publications revealed the importance of genetic predisposition in the etiopathogenesis of TB [5–8]. Single nucleotide polymorphisms (SNPs) in genes as inducible nitric oxide synthase [9, 10], solute carrier protein 11A1 [11], Toll-like receptors [12, 13], nucleotide oligomerization domain 2 [14], CD14 [15], vitamin D nuclear receptor [16], mannose-binding lectin [17], surfactant protein A [18], tumor necrosis factor [19], interleukin (IL)-6 [20] and IL-10 [21], monocyte chemoattractant protein-1 [22], RANTES [23] and C-X-C motif chemokine 10 [24] have been reported [25] to be associated with increased or decreased risk of developing TB.

The complement system plays an important role in host defense against infectious pathogens. It can promote opsonization of pathogens and immune complexes, leukocyte recruitment, inflammation and cell lysis. The lectin pathway is an important pathway of complement activation [26]. Ficolin-2 (also known as P35, L-ficolin) is an innate immunity pattern recognition molecule. It consists of a collagen-like tail and a fibrinogen-related globular head region. A triplet subunit is formed by the collagen-like triple helix, and this then forms higher multimers [27]. Unlike other ficolins, it has complex binding sites within its internal space enabling it to recognize a variety of molecular patterns, such as acetylated sugars and 1,3-β-glucans of pathogens [27]. Ficolin-2 was demonstrated to recognize and bind to a variety of pathogens including hepatitis C virus [28], *pseudomonas aeruginosa* [29], *aspergillus fumigatus* [30], *bovis* [31], and Mtb [32] through MBL-associated serine proteases (MASPs). The pathogen surface is marked with C3b that can induce phagocytosis, formation of the membrane attack complex and destruction of the cell membrane, resulting in direct elimination of the pathogen [33].

Ficolin-2 is encoded by the *FCN2* gene located on chromosome 9q34. The *FCN2* gene has eight exons. Exon 1 encodes the signal peptide and the N-terminal residues. Exon 2 and 3 encode the collagen-like domain. Exon 4 encodes the linker region. Exons 5 to 8 encode the fibrinogen-like domain [34, 35]. SNPs in the *FCN2* gene have been reported in TB and other diseases [35–39], indicating that *FCN2* is involved in infectious diseases. SNPs in the promoter region (-986 G>A, -602 G>A, -557 A>G, -64 A>C and -4 A>G) have been shown to be related to the serum expression of ficolin-2 [40–42]. Two non-synonymous SNPs in exon 8 (+6359 C>T, Thr236Met and +6424 G>T, Ala258Ser) have been demonstrated to enhance or weaken the binding ability of *N*-acetylglucosamine [40, 43]. SNPs of the promoter and exon 8 might also be related to some diseases, such as leprosy [44] hepatitis B [45], malaria [46], schistosomiasis [41], and chronic chagas disease [47]. In addition, SNPs in the *FCN2* gene associated with low levels of ficolin-2 level might predispose an individual to recurrent and/or more severe streptococcal infection [48]. Functional haplotypes that produce normal ficolin-2 levels protect against clinical leprosy [49]. Ficolin-2 insufficiency has been demonstrated to enhance susceptibility to respiratory infections in children [50]. To the best of our knowledge, SNPs in the *FCN2* gene associated with susceptibility to pulmonary TB have not yet been reported. The present study aimed to investigate the association between SNPs of the *FCN2* gene and susceptibility to pulmonary TB in the Chinese Han population. The study provides novel ideas for the prevention and control of pulmonary TB.

## Materials and Methods

### Ethics Statement

The study was approved by the Ethics Committee of School of Medicine (Zhejiang University, China), and informed consent was obtained from all participants before conducting the study. Written informed consent was given by adult participants (or legal guardians of children) for their records to be used in the information system.

### Patients and Controls

A total of 282 pulmonary TB patients (157 males and 125 females; aged 18–70 years, mean age 43 years) were recruited from the Sixth Hospital of Shaoxing (Zhejiang Province, China). The blood samples were collected between January 2012 and May 2014. All TB cases were diagnosed according to the guidelines issued by the Chinese Ministry of Health. All patients met one or more of the following diagnostic criteria: (1) at least one positive sputum smear and/or culture; (2) negative sputum examination, but typical pathology of active TB on chest X-ray; (3) histopathological diagnosis of pulmonary TB; (4) anti-TB drug therapy effective for the suspected TB patients during follow-up observation. Patients with extrapulmonary TB, chronic disease, cancer, autoimmune disease, or HIV infection were excluded. Blood samples were collected from all patients before treatment initiation.

A total of 254 healthy controls (147 males and 107 females; aged 20–72 years, mean age 41 years) were recruited from the Zhejiang Hospital (China). Pulmonary TB, hepatitis B, AIDS, autoimmune diseases and other chronic diseases were excluded after history taking, physical examination, and blood tests.

The demographic characteristics of subjects showed no significant differences between the two groups in age, gender, history of TB, and BCG vaccination. All patients and controls were living in the same geographical region (Southeast China), and were from the same ethnic origin (Chinese Han) (S1 Table).

The early morning fasting blood samples from all participants were collected in EDTA tubes, and then dispensed into sterile centrifuge tubes and stored at -80℃

### Genotyping

*FCN2* SNPs information was obtained from the dbSNP database (http://www.ncbi.nlm.nih.gov/snp/) and HapMap database (http://www.hapmap.org). We selected seven SNPs in the *FCN2* gene: -986 G>A (rs3124952), -602 G>A (rs3124953), -557 A>G (rs3811140), -64 A>C (rs7865453), -4 A>G (rs17514136), +6359 C>T (rs17549193) and +6424 G>T (rs7851696). Distribution and relative positions of 7 SNPs in the *FCN2* gene are shown in S2 Table.

SNPs in the *FCN2* gene were analyzed by PCR amplification and direct sequencing. Genomic DNA was extracted from peripheral blood leukocytes by the DNA extraction kit (QIAamp® DNA Blood Mini Kit, Germany) according to the manufacturer’s instructions. Primer Premier 5.0 software was used to design primers. The following primers were used for the PCR: *FCN2*–986, -557 and -642 sites: forward: TCTCAGGACCACACATCTCCA; reverse: GGTGTGGGCCTTACACAGTA. *FCN2*–64 and -4 sites: forward: AAACCCTTCCTTGTTCCCCG; reverse: AACCTGCCTCGGTTTCCATT. *FCN2* +6359 and +6424 sites: forward: ATGATGATCCTGACCCCTGC; reverse: CCGCACAGCAAGACAAACC. The PCR program consisted of a denaturation at 94℃ for 5 min, followed by 35 cycles of denaturation at 94℃ for 45 s, annealing at 56℃ for 45 s, and extension at 72℃ for 1 min, and a final product extension at 72℃ for 10 min. The amplification products were purified with the PCR purification kit (AxyPrep PCR cleaning kit, USA). The purified products were sequenced in the ABI 3100 sequencer (Applied Biosystems, USA).

### Statistical Analysis

Hardy-Weinberg equilibrium was assessed by using the chi-square test for healthy controls. The chi-square test was used to compare allele and genotype distribution in the pulmonary TB patients and healthy controls by the GraphPad Prism version 5.0 software. The correlation between individual SNP and pulmonary TB was analyzed in five different inheritance patterns (codominant, dominant, recessive, overdominant, and log-additive) using the logistic regression method. Odds ratios (OR) and 95% confidence intervals (CI) were calculated by the Miettinen method, and *P* < 0.05 was considered statistically significant. Haplotype frequencies and associations were calculated by Haploview version 4.2, which uses the expectation-maximization algorithm. Pairwise linkage disequilibrium was estimated by calculating pairwise D' and r^2^. The nonparametric Mann-Whitney U test was used to analyze continuous variables of different groups.

## Results

### Allele Frequencies and Genotypes Frequencies

**Fig 1:**
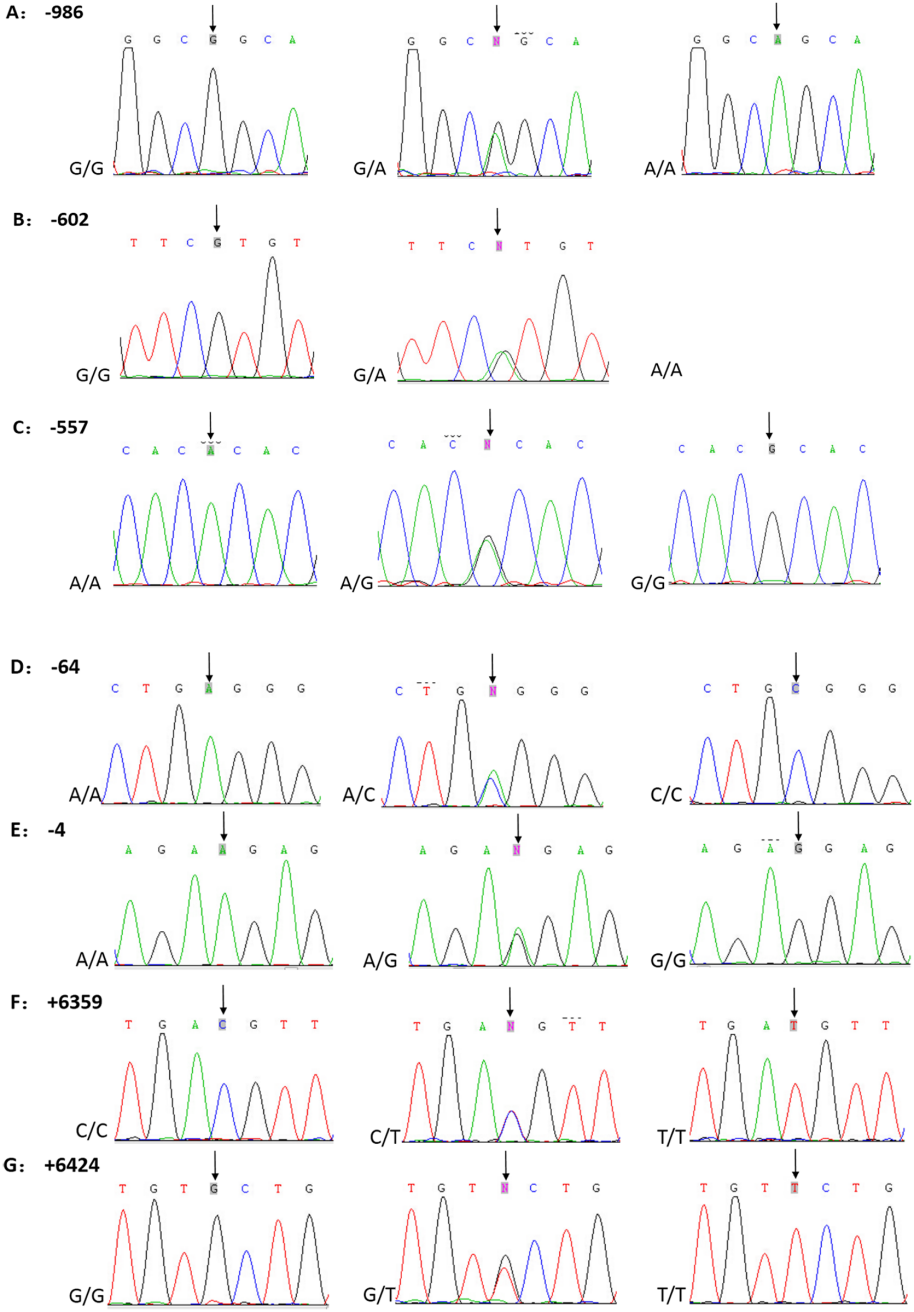
The DNA sequences with 7 SNPs in the *FCN2* gene.

Seven SNPs (-986 G>A, -602 G>A, -557 A>G, -64 A>C, -4 A>G, +6359 C>T, and +6424 G>T) in the *FCN2* gene were sequenced after PCR amplification (Fig 1). The allelic distributions of *FCN2* SNPs were in accordance with Hardy-Weinberg equilibrium in the control group (*P* > 0.05) (Table 1). There were no significant differences in allele frequencies between the pulmonary TB group and the healthy controls group (*P* > 0.05) (Table 1).

**Table 1:** Distribution of the *FCN2* SNP allele frequencies and genotype frequencies in the pulmonary TB group (n = 282) and the control group (n = 254).

SNP sites	Allele	Controls N(Freq)	Patients N(Freq)	*P* Value	OR	95% CI	Genotype	Controls N(Freq)	Patients N(Freq)	*P* Value	OR	95% CI
-986 G/A	G	475(0.939)	530(0.940)				GG	224(0.885)	250(0.887)		1	
	A	31(0.061)	34(0.060)	0.947	0.983	0.595–1.624	GA	27(0.107)	30(0.106)	0.987	0.996	0.574–1.726
	HWE(*P*)	0.251					AA	2(0.008)	2(0.007)	0.913	0.896	0.125–6.417
-602 G/A	G	500(0.988)	557(0.988)				GG	247(0.976)	275(0.975)		1	
	A	6(0.012)	7(0.012)	0.934	1.047	0.350–3.138	GA	6(0.024)	7(0.025)	0.934	1.048	0.347–3.161
	HWE(*P*)	0.849					AA	0	0			
-557 A/G	A	403(0.796)	456(0.809)				AA	160(0.632)	177(0.628)		1	
	G	103(0.204)	108(0.191)	0.62	0.927	0.686–1.253	AG	83(0.328)	102(0.362)	0.567	1.111	0.775–1.593
	HWE(*P*)	0.851					GG	10(0.040)	3(0.010)	0.037	0.271	0.073–1.003
-64 A/C	A	399(0.795)	452(0.804)				AA	158(0.629)	174(0.619)		1	
	C	103(0.205)	110(0.196)	0.701	0.943	0.698–1.273	AC	83(0.331)	104(0.370)	0.482	1.138	0.793–1.631
	HWE(*P*)	0.826					CC	10(0.040)	3(0.011)	0.038	0.272	0.074–1.008
-4 A/G	A	476(0.948)	535(0.952)				AA	226(0.900)	255(0.907)		1	
	G	26(0.052)	27(0.048)	0.779	0.924	0.532–1.606	AG	24(0.096)	25(0.089)	0.79	0.923	0.513–1.662
	HWE(*P*)	0.675					GG	1(0.004)	1(0.004)	0.932	0.886	0.055–14.260
+6359 C/T	C	485(0.955)	537(0.952)				CC	232(0.913)	256(0.908)		1	
	T	23(0.045)	27(0.048)	0.84	1.06	0.600–1.874	CT	21(0.083)	25(0.089)	0.806	1.079	0.588–1.979
	HWE(*P*)	0.487					TT	1(0.004)	1(0.003)	0.945	0.906	0.056–14.580
+6424 G/T	G	405(0.797)	456(0.809)				GG	162(0.638)	177(0.628)		1	
	T	103(0.203)	108(0.191)	0.643	0.931	0.689–1.259	GT	81(0.319)	102(0.362)	0.441	1.153	0.803–1.654
	HWE(*P*)	0.829					TT	11(0.043)	3(0.010)	0.024	0.25	0.068–0.911

SNP: single nucleotide polymorphism; HWE: Hardy-Weinberg Equilibrium; N: numbers; Freq: frequency; OR: odds ratios; 95% CI: 95% confidence intervals; *P* value and odd ratio were obtained by Chi-square test.

The frequency of GG genotype at -557 A>G site in the pulmonary TB group (0.010) was lower than the control group (0.040), and there was a significant difference between the two groups (*P* = 0.037; OR = 0.271; 95% CI, 0.073–1.003). The frequency of CC genotype at -64 A>C site was also lower in the pulmonary TB group (0.011) than the control group (0.040), and there was a significant difference between the two groups (*P* = 0.038; OR = 0.272; 95% CI, 0.074–1.008). The +6424 G>T TT genotype also had lower frequency in the pulmonary TB group (0.010) than the control group (0.043), and there was a significant difference between the two groups (*P* = 0.024; OR = 0.250; 95% CI, 0.068–0.911) (Table 1).

### Correlation Analysis

To correlate the ficolin-2 levels with their respective genotypes, we went through the previous publications and performed genotype-phenotype correlation analysis. We found that -557 A>G, -64 A>C, and +6424 G>T were associated with lower ficolin-2 levels, while -986 G>A, -602 G>A, -4 A>G, and +6359 C>T corresponded to higher ficolin-2 levels (Table 2).

**Table 2:** Significant changes in Ficolin-2 levels correlate with *FCN2* SNPs in previous publications.

	Ficolin-2 levels						
Publications	-986 G>A	-602 G>A	-557 A>G	-64 A>C	-4 A>G	+6359 C>T	+6424 G>T
Hummelshoj *et al*. [40]	Higher	Higher	NS	NS	Higher	ND	ND
Kilpatrick *et al*. [42]	Higher	Higher	Lower	Lower	Higher	Higher	Lower
Tong *et al*. [45]	Higher	NS	ND	ND	NS	ND	Lower
Faik *et al*. [46]	NS	NS	ND	ND	NS	ND	Lower
Fog *et al*. [62]	Higher	Higher	NS	NS	Higher	NS	Lower
Metzger *et al*. [63]	Higher	Higher	ND	ND	NS	NS	Lower
Cedzynski *et al*. [64]	ND	ND	ND	Lower	NS	NS	Lower

SNP: single nucleotide polymorphism; NS: not significant; ND: not determined.

The correlation between SNPs and pulmonary TB was analyzed using the logistic regression method. After adjustment for age and gender, the -557 A>G site was found to be the recessive model (*P* = 0.02; OR = 0.24; 95% CI, 0.07–0.89), and was in significant association with pulmonary TB. The -64 A>C site was found to be the recessive model (*P* = 0.02; OR = 0.24; 95% CI, 0.07–0.89) and codominant model (*P* = 0.047), and was in significant association with pulmonary TB. The +6424 G>T site was found to be the recessive model (*P* = 0.01; OR = 0.23; 95% CI, 0.06–0.82) and codominant model (*P* = 0.03), and was in significant association with pulmonary TB. Other sites were not significantly correlated with pulmonary TB in five different inheritance patterns (*P* > 0.05). According to the minimum Akaike Information Criterion (AIC), the best genetic models were the recessive models for -557 A>G, -64 A>C, and +6424 G>T (S3 Table).

### Linkage Disequilibrium and Haplotypes

**Fig 2:**
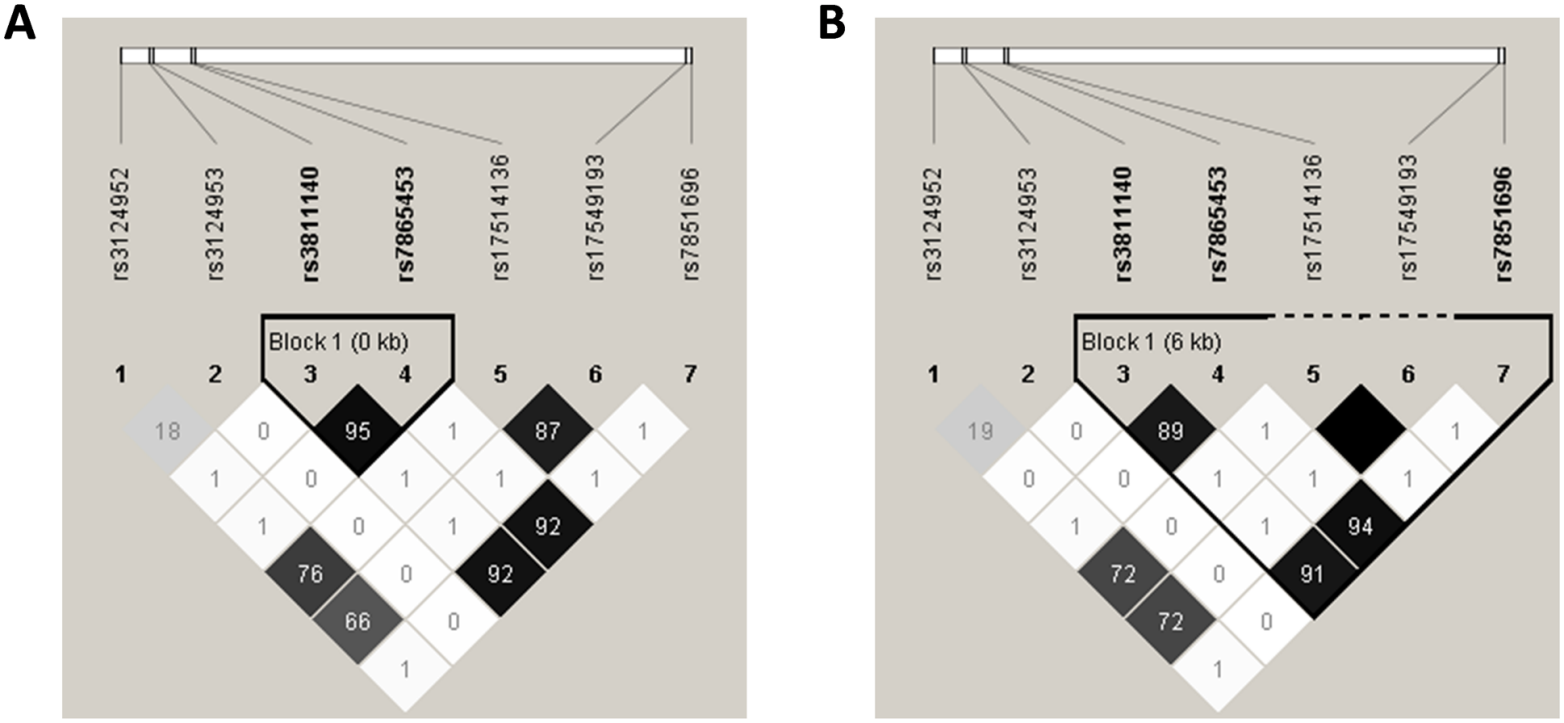
Haploview plot illustrating the linkage disequilibrium (LD) of the FCN2 variants. **A**: Linkage disequilibrium of 7 functional *FCN2* single nucleotide polymorphism (SNPs) in the healthy controls. Block 1 represent the 2 SNPs (−557A>G and −64 A>C) completely linked. **B**: Linkage disequilibrium of 7 functional *FCN2* SNPs in the pulmonary TB group. Block 1 represent the 3 SNPs (−557A>G, −64 A>C and +6424 G>T) completely linked. Open squares indicate a high degree of LD (LD coefficient D′ = 1) between pairs of markers. Numbers indicate the r^2^ value.

There were strong linkage disequilibrium (r^2^>0.80, *P* < 0.0001) between 7 SNPs of the *FCN2* gene except the -602 G>A site. The -557 A>G and -64 A>C sites were completely linked in the control group, while the -557 A>G, -64 A>C and +6424 G>T sites were completely linked in the pulmonary TB group (Fig 2). The linkage disequilibrium maps were basically identical in the two groups.

Based on logistic regression analysis, we observed the correlations between the constructed haplotypes and the occurrence of pulmonary TB. Considering the strong linkage disequilibrium between -557 A>G, -64 A>C and +6424 G>T sites, and -4 A>G and +6359 C>T sites, we chose -986 G>A, -602 G>A, +6359 C>T, and +6424 G>T sites for haplotype analysis. The frequencies of GGCG and AGTG haplotypes in the pulmonary TB group were higher than the control group, while the frequency of GGCT and AACG haplotype in the pulmonary TB group were lower than the control group. However, all haplotypes with the *FCN2* gene polymorphisms were not significantly correlated with pulmonary TB (*P* > 0.05) (Table 3).

**Table 3:** Haplotype frequencies of polymorphisms variants of the -986 G>A, -602 G>A, +6359 C>T and +6424 G>T SNPs in patients with pulmonary TB and healthy controls.

	Allele at marker							
Haplotype	-986	-602	+6359	+6424	Controls Freq	Patients Freq	*P* value^a^	OR (95% CI)
1	G	G	C	G	0.736	0.752	0.603	1.077 (0.815–1.422)
2	G	G	C	T	0.200	0.186	0.547	0.911 (0.672–1.235)
3	A	G	T	G	0.043	0.046	0.867	1.051 (0.587–1.882)
4	A	A	C	G	0.011	0.009	0.830	0.877 (0.263–2.918)
Global haplotype association *P* value: 0.934								

^a^ Adjusted for age and sex.

Freq: frequency of haplotype; OR: odds ratios; 95% CI: 95% confidence intervals.

## Discussion

Previous studies in our laboratory (S4 Table) showed that SNPs in genes were associated with susceptibility to pulmonary TB [11, 14, 51–56]. The present study is the first to describe the correlation between seven SNPs (-986 G>A, -602 G>A, -557 A>G, -64 A>C, -4 A>G, +6359 C>T, and +6424 G>T) in the *FCN2* gene and pulmonary TB. We found significant lower frequencies of -557 A>G GG genotype, -64 A>C CC genotype, and +6424 G>T TT genotype in TB patients (Table 1), suggesting the protective role of these SNPs. There were strong linkage disequilibrium between 7 SNPs except the -602 G>A site. Moreover, -557 A>G, -64 A>C, and +6424 G>T, and -4 A>G and +6359 C>T sites were linked in TB patients. However, there were no significantly correlated haplotypes.

We found that the heterozygote frequency of -602 G>A appeared relatively lower, and there was no AA homozygous genotype. This is consistent with the studies conducted on the Vietnamese [35], Nigerian [35], and Japanese populations [57]. However, the frequencies of GA genotype in the Brazilian and European populations have been found to be much higher than the above mentioned populations. Therefore, geographical differences of the -602 G>A genotype frequencies in this study may be the result of racial evolution and environmental interactions. The frequencies of GG, GT and TT genotypes at +6424 G>T in the present study are consistent with the frequencies found in the Brazilian, Nigerian, Vietnamese, and European populations [35].

The frequency of -557 A>G GG genotype in the pulmonary TB group was lower than the control group. However, previous research on the association between the *FCN2* gene and leprosy [44] has shown that the frequency of -557 A>G GG genotype in patients with leprosy was not significantly different (*P* = 0.085) compared to the healthy controls. In the present study, -64 A>C CC genotype was a protective factor in pulmonary TB patients. However, no such findings were observed in patients with Behcet's disease [57], indicating that *FCN2* polymorphisms vary with different diseases. The frequency of +6424 G>T TT genotype also showed different function of *FCN2* between pulmonary TB and paucibacillary leprosy [44].

Whole blood RNA has been widely applied to characterize differences between individuals with active TB [58]. Mtb evades the host immune system by being phagocytosed by macrophages and neutrophils [59]. Complement-dependent opsonisation of extracellular mycobacteria may assist Mtb to enter into macrophages [31, 60]. Serum mannan binding lectin (MBL) and ficolin-2 have been shown to directly bind to the Mtb, leading to MASP-2 activation [31]. Luo et al. [32] found that ficolin-2 could recognize and bind to the surface glycolipid of virulent Mtb H37Rv. Opsonophagocytosis has also been shown to be promoted by ficolin-2. Herpers et al. [61] found that +6424 G>T is a coding SNP located in exon 8, leading to amino acid substitutions within the fibrinogen-like (FBG) domain. FBG domain is related to the binding ability of ficolin-2. In addition, +6424 G>T T allele could enhance the binding ability for *N*-acetylglucosamine [43]. Through previous publications, we found that -557 A>G, -64 A>C, and +6424 G>T are associated with lower ficolin-2 levels (Table 2) [40, 42, 45, 46, 62–64]. As -557 A>G, -64 A>C, and +6424 G>T were linked in pulmonary TB patients, therefore, we assumed that this may affect the ficolin-2 expression and the binding activity of pathogens by influencing the binding ability of *N*-acetylglucosamine, ultimately affecting the activation of complements. The activity of the complement system depends on the genetic integrity of the genes. Thus gene mutation, SNPs in particular, may play an important role in the immune process.

The present study found that the variant homozygous genotype of -557 A>G, -64 A>C and +6424 G>T in the *FCN2* gene may be protective factors for TB, and were significantly associated with TB in the recessive model. Correlation between *FCN2* SNPs and pulmonary TB revealed an important role of *FCN2* in the pathogenesis of TB. This study provides a novel idea for prevention and control of TB from a genetics perspective.

## Supporting Information

S1 Table Demographic Characteristics of Subjects. (DOC)

S2 Table Location of 7 SNPs in the *FCN2* gene. (DOC)

S3 Table Association of -557 A>G, -64 A>C, and +6424 G>T with pulmonary TB using logistic regression. (DOC)

S4 Table Studies on the susceptibility genes for pulmonary TB in our laboratory. (DOC)
