# Supplementary material for: Association of the FCN2 Gene Single Nucleotide Polymorphisms with Susceptibility to Pulmonary Tuberculosis
Source: PLoS One. 2015 Sep 17;10(9):e0138356. doi: 10.1371/journal.pone.0138356 (PMC4574923; doi:10.1371/journal.pone.0138356)
Supplement: S1 Table — (DOC) [file pone.0138356.s001.doc]

**Supporting Information**

**S1 Table.** **Demographic Characteristics of Subjects.**

| Variables | TB patients (N=282) | Healthy controls (N=254) | *P* value |
| --- | --- | --- | --- |
| Age, years range (mean ± SD) | 18-70(43.1±17.2) | 20-72(40.9±11.0) | 0.215 ^a^ |
| Gender: female, no. (%) | 125(44.3) | 107(42.1) | 0.665^b^ |
| Tuberculin skin test (>10 mm), no. (%) | 183(64.9) | ND | / |
| Sputum culture-proven, no. (%) | 166(58.9) | ND | / |
| Family history of TB, no. (%) | 25(8.86) | 13(5.12) | 0.092^b^ |
| BCG vaccination, no. (%) | 153(54.2) | 128(50.4) | 0.371^b^ |
| Current Smoker, no. (%) | 94(33.3) | ND | / |
| Cavitary lesion, no. (%) | 90(31.9) | ND | / |

TB: tuberculosis; N: number of subjects; ND: not determined. ^a^*P* value between TB patients and healthy controls, for *t* test. ^b^*P* value between TB patients and healthy controls, for χ^2^ test.
