# Supplementary material for: Association of the FCN2 Gene Single Nucleotide Polymorphisms with Susceptibility to Pulmonary Tuberculosis
Source: PLoS One. 2015 Sep 17;10(9):e0138356. doi: 10.1371/journal.pone.0138356 (PMC4574923; doi:10.1371/journal.pone.0138356)
Supplement: S2 Table — (DOC) [file pone.0138356.s002.doc]

**Supporting Information**

**S2 Table. Location of 7 SNPs in the *FCN2* gene.**

| Position | Major allele | Minor allele | dbSNP ID | Amino acid change | Region |
| --- | --- | --- | --- | --- | --- |
| -986 | G | A | rs3124952 |  | *FCN2* Promoter |
| -602 | G | A | rs3124953 |  | *FCN2* Promoter |
| -557 | A | G | rs3811140 |  | *FCN2* Promoter |
| -64 | A | C | rs7865453 |  | *FCN2* Promoter |
| -4 | A | G | rs17514136 |  | *FCN2* Promoter |
| +6359 | C | T | rs17549193 | Thr236Met | *FCN2* Exon 8 |
| +6424 | G | T | rs7851696 | Ala258Ser | *FCN2* Exon 8 |
