# Supplementary material for: Association of the FCN2 Gene Single Nucleotide Polymorphisms with Susceptibility to Pulmonary Tuberculosis
Source: PLoS One. 2015 Sep 17;10(9):e0138356. doi: 10.1371/journal.pone.0138356 (PMC4574923; doi:10.1371/journal.pone.0138356)
Supplement: S3 Table — (DOC) [file pone.0138356.s003.doc]

**Supporting Information**

**S3 Table.** **Association of -557 A>G, -64 A>C, and +6424 G>T with pulmonary TB using logistic regression.**

| Model^a^ | Genotype | Controls(Freq) | Patients(Freq) | OR (95% CI) | *P* value | AIC | BIC |
| --- | --- | --- | --- | --- | --- | --- | --- |
| **-557 A>G** |  |  |  |  |  |  |  |
| Codominant | A/A | 160 (0.632) | 177 (0.628) | 1 | 0.05 | 738.4 | 759.8 |
|  | A/G | 83 (0.328) | 102 (0.362) | 1.12 (0.78-1.60) |  |  |  |
|  | G/G | 10 (0.040) | 3 (0.010) | 0.25 (0.07-0.93) |  |  |  |
| Dominant | A/A | 160 (0.632) | 177 (0.628) | 1 | 0.92 | 742.2 | 759.3 |
|  | A/G-G/G | 93 (0.368) | 105 (0.372) | 1.02 (0.72-1.45) |  |  |  |
| Recessive | A/A-A/G | 243 (0.960) | 279 (0.989) | 1 | 0.02 | 736.7 | 753.9 |
|  | G/G | 10 (0.040) | 3 (0.011) | 0.24 (0.07-0.89) |  |  |  |
| Overdominant | A/A-G/G | 170 (0.672) | 180 (0.638) | 1 | 0.39 | 741.5 | 758.6 |
|  | A/G | 83 (0.328) | 102 (0.362) | 1.17 (0.82-1.68) |  |  |  |
| Log-additive | --- | --- | --- | 0.91 (0.66-1.25) | 0.57 | 741.9 | 759.0 |
| **-64 A>C** |  |  |  |  |  |  |  |
| Codominant | A/A | 158 (0.629) | 174 (0.619) | 1 | 0.05 | 733.6 | 754.9 |
|  | A/C | 83 (0.331) | 104 (0.370) | 1.16 (0.80-1.66) |  |  |  |
|  | C/C | 10 (0.040) | 3 (0.011) | 0.25 (0.07-0.94) |  |  |  |
| Dominant | A/A | 158 (0.629) | 174 (0.619) | 1 | 0.77 | 737.6 | 754.7 |
|  | A/C-C/C | 93 (0.371) | 107 (0.381) | 1.05 (0.74-1.50) |  |  |  |
| Recessive | A/A-A/C | 241 (0.960) | 278 (0.989) | 1 | 0.02 | 732.2 | 749.3 |
|  | C/C | 10 (0.040) | 3 (0.011) | 0.24 (0.07-0.89) |  |  |  |
| Overdominant | A/A-C/C | 168 (0.669) | 177 (0.630) | 1 | 0.30 | 736.6 | 753.7 |
|  | A/C | 83 (0.331) | 104 (0.370) | 1.21 (0.85-1.74) |  |  |  |
| Log-additive | --- | --- | --- | 0.94 (0.68-1.29) | 0.69 | 737.5 | 754.6 |
| **+6424 G>T** |  |  |  |  |  |  |  |
| Codominant | G/G | 162 (0.638) | 177 (0.628) | 1 | 0.03 | 738.8 | 760.2 |
|  | G/T | 81 (0.319) | 102 (0.362) | 1.17 (0.81-1.68) |  |  |  |
|  | T/T | 11 (0.043) | 3 (0.010) | 0.24 (0.06-0.87) |  |  |  |
| Dominant | G/G | 162 (0.638) | 177 (0.628) | 1 | 0.78 | 743.7 | 760.8 |
|  | G/T-T/T | 92 (0.362) | 105 (0.372) | 1.05 (0.74-1.50) |  |  |  |
| Recessive | G/G-G/T | 243 (0.957) | 279 (0.989) | 1 | 0.01 | 737.5 | 754.6 |
|  | T/T | 11 (0.043) | 3 (0.011) | 0.23 (0.06-0.82) |  |  |  |
| Overdominant | G/G-T/T | 173 (0.681) | 180 (0.638) | 1 | 0.27 | 742.5 | 759.7 |
|  | G/T | 81 (0.319) | 102 (0.362) | 1.23 (0.86-1.76) |  |  |  |
| Log-additive | --- | --- | --- | 0.93 (0.68-1.27) | 0.63 | 743.6 | 760.7 |

^a^Adjusted for age and sex. OR: odds ratios; 95% CI: 95% confidence intervals; AIC: Akaike’s Information Criterion; BIC: Bayesian Information Criterion.
