# Supplementary material for: Association of the FCN2 Gene Single Nucleotide Polymorphisms with Susceptibility to Pulmonary Tuberculosis
Source: PLoS One. 2015 Sep 17;10(9):e0138356. doi: 10.1371/journal.pone.0138356 (PMC4574923; doi:10.1371/journal.pone.0138356)
Supplement: S4 Table — (DOC) [file pone.0138356.s004.doc]

**Supporting Information**

**S4 Table. Studies on the susceptibility genes for pulmonary TB in our laboratory.**

| Gene | Position | Relationship with TB | Date |
| --- | --- | --- | --- |
| *CD14* | G-1145A, C-159T in promoter | Protective factor for TB | Zhao *et al*. Genet Mol Res. 2012 Sep 25;11(3):3425-31. |
| *MD-2* | C-1625G, C-1201G, G-1174T, A-1064G, T-538G, and T-475A in promoter | Lack of association | Xue *et al*. Genet Mol Res. 2010 Aug 17;9(3):1584-90. |
| *TLR2* | the microsatellite (GT)n polymorphisms in intron 2 | Susceptibility to TB | Xue *et al*. Clin Chem Lab Med. 2010 Jun;48(6):785-9. |
|  | Arg677Trp, Arg753Gln in the exon | Lack of association | Xue *et al*. Int J Immunogenet. 2010 Apr;37(2):135-8. |
| *TLR4* | Asp299Gly, Thr399Ile in the exon | Lack of association | Xue *et al*. Int J Immunogenet. 2010 Apr;37(2):135-8. |
| *TIRAP* | G286A in exon 5 | Susceptibility to TB | Zhang *et al*. Genet Mol Res. 2011 Jan 4;10(1):7-15. |
| *CTLA4* | A+49G in the exon 1 | Protective factor for TB | Wang *et al*. Int J Biol Sci. 2012;8(7):945-52. |
| *NOD2* | Arg587Arg in the exon 4 | Susceptibility to TB | Zhao *et al*. BMC Infect Dis. 2012 Apr 14;12:91. |
| *MRCI* | G1186A in the exon 7 | Susceptibility to TB | Zhang *et al*. Int J Biol Sci. 2012;8(3):375-82. |
